# Playing Level and Position Differences in Body Characteristics and Physical Fitness Performance Among Male Team Handball Players

**DOI:** 10.3389/fbioe.2019.00149

**Published:** 2019-06-21

**Authors:** Souhail Hermassi, Kevin Laudner, René Schwesig

**Affiliations:** ^1^Sport Science Program, College of Arts and Sciences, Qatar University, Doha, Qatar; ^2^School of Kinesiology and Recreation, Illinois State University, Normal, IL, United States; ^3^Department of Orthopaedic and Trauma Surgery, Martin-Luther-University Halle-Wittenberg, Halle, Germany

**Keywords:** playing position, physical performance, elite players, handball team, throwing ability

## Abstract

The primary aim of the study was to examine the anthropometric characteristics, as well as the physical performance of professional handball players classified by playing position and competition level. Twenty male players (age: 20.4 ± 0.88 years) from the first handball league and 18 male players (age: 21.3 ± 1.61 years) from the second handball league were categorized as backs (8/8), pivots (5/4), and wings (7/6). The following variables were measured in both groups: peak power; vertical squat jump (SJ), and countermovement jump (CMJ); sprint times over 15 and 30 m; handball throwing velocity during the jump shot (JS); and 3 steps shot (T3 step); upper and lower limb muscle volumes; Yo-Yo Intermittent Recovery Test. Anthropometric data revealed significantly (*p* = 0.003, $\eta_{\text{p}}^{2}$ = 0.248) less muscle volume for second league players (3.13 ± 0.29 l) than for first league (3.71 ± 0.82 l). The cross-sectional area for the first league players was also larger (*p* = 0.010, $\eta_{\text{p}}^{2}$ = 0.192). Regarding performance parameters, we found significant (*p* < 0.05 and $\eta_{\text{p}}^{2}$ > 0.20) league differences in five of 15 (33%) performance parameters: running throw ($\eta_{\text{p}}^{2}$ = 0.285), SJ power ($\eta_{\text{p}}^{2}$ = 0.670), SJ velocity ($\eta_{\text{p}}^{2}$ = 0.900), peak upper limb power ($\eta_{\text{p}}^{2}$ = 0.231) and Yo-Yo-IR 1 ($\eta_{\text{p}}^{2}$ = 0.348). The second league players showed higher SJ velocity than the first league players ($\eta_{\text{p}}^{2}$ = 0.900). In contrast, we detected a greater difference in SJ power ($\eta_{\text{p}}^{2}$ = 0.670) but in favor of the first league players. Pivots were the players with the highest throwing velocity and wings were the fastest (15, 30 m sprint), strongest (countermovement jump), and most enduring (Yo-Yo-IR 1) athletes. Backs showed consistently the lowest level throwing velocity and sprint performance (exception: second league). The anthropometric differences between playing levels and playing positions may indicate the advantageous characteristics that the respective position demands, whereas the playing position differences in physical fitness characteristics may indicate training specificity issues that must be addressed cautiously.

## Introduction

Team handball is classified as a high-intensity, body-contact sport that demands a high level of aerobic and anaerobic fitness (Hermassi et al., 2017). Several studies have reported that, in handball players, in addition to the technical skills and tactics, the anthropometric characteristics, and high levels of force, power, and throwing velocity constitute the determining factors for competitive success (Gorostiaga et al., 2006; Karcher and Buchheit, 2014; Wagner et al., 2016; Fieseler et al., 2017;Hermassi et al., 2017).

Few studies have compared the anthropometric and physical characteristics for handball players of different levels and different playing positions. Although some studies have analyzed some physiological characteristics of elite handball players, little information is available concerning the physical (e.g., sprint, throwing performance) and anthropometric characteristics of current professional handball players. Examination of fitness profiles could be of great importance for optimal construction of training regimens to improve handball performance and in the orthopedic care of such players.

The profiling of players can be a valuable tool when identifying talent, determining strengths and weaknesses, assigning playing positions, and optimizing the design of strength and conditioning training programs (Karcher and Buchheit, 2014; Fieseler et al., 2017; Schwesig et al., 2017). Some may anticipate that professional handball players who play indoors on a small court might show greater homogeneity across playing positions than competitors who play on larger fields such as soccer (Karcher and Buchheit, 2014). Nevertheless, several studies of handball players have reported significant differences among playing positions for various physiological, physical, and anthropometric characteristics (Srhoj et al., 2002; Šibila and Pori, 2009; Zapartidis et al., 2011; Rousanoglou et al., 2014; Fieseler et al., 2017; Schwesig et al., 2017).

Anthropometric parameters and physical and motor test have been identified as fundamental in order to determine the success of the performance in handball (Karcher and Buchheit, 2014; Fieseler et al., 2017; Schwesig et al., 2017). Thus, some of the previous studies have provided the specific performance measures that could be the most useful (Šibila and Pori, 2009; Karcher and Buchheit, 2014; Fieseler et al., 2017). Regarding anthropometry, one study demonstrated that body composition could have an influence in the game's performance, namely a larger hand size or greater handgrip strength creates greater control of the ball, and a larger wingspan creates a higher occupation of space in defensive and offensive actions (Karcher and Buchheit, 2014). Granados et al. (2013) showed that the higher values of fat-free mass resulted in a higher performance, especially because of the increase in the muscular power and strength. On the other hand, one study evaluated different basic motor skills as decisive performance factors, showing that the fine motor skills in the upper limbs could be essential for performance (Srhoj et al., 2002).

There are few studies that have collectively evaluated the physical condition, anthropometric profile, muscular power, and throwing velocity in handball players (Srhoj et al., 2002; Šibila and Pori, 2009; Zapartidis et al., 2011; Rousanoglou et al., 2014; Fieseler et al., 2017; Schwesig et al., 2017). This lack of research is even more important among handball players of different levels because there are fewer studies that have been published (Fieseler et al., 2017; Schwesig et al., 2017), and furthermore, none of them have focused on studying the throwing velocity of the players. Although some studies have analyzed physiological characteristics of elite handball players, little information is available concerning the physical (e.g., strength, peak power sprint, and throwing performance) and anthropometric characteristics (e.g., muscle volume of upper and lower limb) of current professional handball players. Examination of fitness profiles could be of great importance for optimal construction of training regimens to improve handball performance and in the orthopedic care of these players.

The purpose of this study was 2-fold. The primary aim was to profile and compare the physical and performance characteristics of first and second-league male handball players. The secondary aim was to compare these same characteristics between playing positions. We hypothesized that throwing, sprinting, muscle power, jumping, and aerobic performance are significantly different between first and second league players, as well as between playing positions.

## Materials and Methods

### Participants

All procedures were approved by the Institutional Review Committee [Research Unit Sport Performance, Health, and Society: University of La Manouba] for the ethical use of human subjects, according to current national and international laws and regulations. Twenty premier league professional male team-handball players (mean ± SD; age: 20.4 ± 0.88 years, body mass: 87.5 ± 12.3 kg, body height: 1.84 ± 0.08 m) and 18 second league male professional team-handball players (mean ± SD; age: 21.3 ± 1.51 years, body mass: 86.9 ± 12.2 kg, body height: 1.85 ± 0.07 m) voluntary agreed to participate in this study. All athletes [9 pivots (five first league, four second league), 13 wings (seven first league, six second league), 16 backs (eight first league, eight second league)] were asymptomatic and had passed medical examinations prior to inclusion and participation in the study. Participants gave their written informed consent after receiving both a verbal and a written explanation of the experimental design and its potential risks. Subjects were free to withdraw from the study without penalty at any time.

### Experimental Design

This study examined if anthropometric and physical fitness parameters are different between male handball players of different competitive levels and playing positions. Two distinct groups of handball players were identified consisting of players from the first and second leagues. The subjects were carefully familiarized with the testing protocol, as they had been previously tested on several occasions in season for training prescription purposes. All of the players within a given team were assessed on the same day, and the tests were performed in the same order.

All tests were conducted in the middle of the pre-seasonal training and ahead of the competitive season. Furthermore, testing sessions were carried out at the same time of the day, and under the same experimental conditions, at least 3 days after the most recent competition. To reduce the influence of uncontrolled variables, all participants were instructed to maintain their typical lifestyle and diet habits before and during the study. Players maintained their normal intake of food and fluids, but abstained from physical exercise for 1 day before testing. They also drank no caffeine-containing beverages for 4 h before testing, and ate no food for 2 h before testing. Strong verbal encouragement was provided to all subjects to promote maximal effort throughout testing.

#### Anthropometry

##### The upper limbs muscle volume

The muscle volume of the upper limbs was estimated as detailed previously, using circumferences and skin-fold thicknesses measured at different levels of the arm and the forearm, the length of the upper limb, and the breadth of the humeral condyles (Jones and Pearson, 1969; Shephard et al., 1988a,b).

Muscle volumes were estimated as:

$$\begin{array}{r} \text{Muscle volume}=\text{total limb volume}\\ -(\text{fat volume}+\text{bone volume}) \end{array}$$

The total limb volume was estimated as the volume of a cylinder, based on its length (L), corresponding to the distance from the acromion to the minimum wrist circumference, and the mean of five limb circumferences (axilla, maximum relaxed biceps, just proximal to the elbow, maximum over the relaxed forearm, and minimum above the styloid process) according to the formula:

$$\begin{array}{c} \text{Total limb volume}=(\Sigma {\text{C}}^{2})•\text{L/}62.8 \end{array}$$

where ΣC^2^ is the sum of the squares of the five circumferences of the corresponding limb.

Skin folds were assessed using a standard Harpenden caliper (Baty International, Burgess Hill, Sussex, UK). The fat volume was calculated as:

$$\begin{array}{c} (\Sigma \text{C}/5)•(\Sigma \text{S}/2\text{n})\text{L} \end{array}$$

where ΣS is the sum of three skin folds for the upper limb (biceps, triceps, and mid-forearm), and “n” represents the number of skin folds measured on each limb.

Bone volume was calculated as:

$$\begin{array}{r} \text{π}∙{\left(\text{F}∙\text{D}\right)}^{2}∙\text{L} \end{array}$$

where D is the humeral intercondylar diameter, F is a geometric factor (0.21 for the upper limb), and L is the limb length as measured above.

Standard equation equations were used to predict the percentage of body fat from the biceps, triceps, subscapular, and suprailiac skinfold readings (Vandewalle et al., 1987):

$$\begin{array}{c} \%Body\;fat=a.\;log\;(\sum 4\;folds)-b \end{array}$$

where ΣS is the sum of the four skinfold readings (in mm), and *a* and *b* are constants dependent on sex and age.

##### Leg muscle volume

Circumferences and skin-fold thickness at different levels of the thigh and the calf, the length of the leg and the breadth of the knee condyles were measured to estimate the leg muscle volume.

$$\begin{array}{r} \text{Muscle volume}=\text{total limb volume}\\ -(\text{fat volume}+\text{bone volume}) \end{array}$$

The total limb volume was estimated as the volume of a cylinder determined by: the distance (L) from the trochanter major to the external malleolar of the ankle. The basal area of the cylinder was based on the mean area of five limb circumferences (C) (maximal thigh, mid-thigh, just below the patella, maximal calf, and just above the ankle).

$$\begin{array}{c} \text{Total limb volume}=(\sum {\text{C}}^{2})•\text{L}/62.8 \end{array}$$

where ∑C^2^ is the sum of the squares of the five circumferences.

$$\begin{array}{c} \text{Fat volume}=(\sum \text{C}/5)•(\sum \text{S}/2\text{n})\text{L} \end{array}$$

where ∑S is the sum of four skinfolds (front to the mid-thigh, back of to the mid-thigh, back of calf, and outside of calf), as determined with a Holtain skin fold caliper and where n is the number of skinfolds measured.

$$\begin{array}{c} \text{Bone volume}=\pi •(\text{F}•\text{D})2•\text{L} \end{array}$$

where D is the femoral intercondylar diameter and F is a geometrical factor (equal to 0.235 for the leg, implying that the bone radius is 23.5% of the femoral intercondylar diameter). The accuracy of this anthropometrical method was previously validated by comparison with dual-energy x-ray absorptiometry (*r* = 0.94; *p* < 0.01) (Chelly et al., 2006).

##### Mean cross sectional area (CSA) of the thigh

The mean thigh CSA was calculated from the maximal and mid-thigh circumferences, after deduction of the appropriate skin-fold thicknesses:

$$\begin{array}{rcl} \text{Circumference }\left(\text{C}\right) & = & 2\pi ∙\text{Radius}\left(\text{R}\right)\\ \text{R} & = & \text{C}/2\text{π} \end{array}$$

R is thus the radius of a transverse section of the muscular mid-thigh, after deduction of the thickness of the overlying skin-folds.

$$\begin{array}{r} \text{r}=\text{R}-[(\text{mid}-\text{thigh anterior skin fold}+\text{mid}\\ -\text{thigh posterior skin fold})/4] \end{array}$$

##### Squat jump and countermovement jump

Prior to jump testing participants followed a general warm-up procedure that included 5 min of cycling with a 60 W load, stretching of lower limbs muscles (gastrocnemious, quadriceps, hip flexors, hamstrings, and gluteals) and 2 min of jumping exercises. Players performed the stretching exercises twice holding each stretch for 15 s and alternating between each leg in order to give adequate recovery before the next repetition. Jumping exercises included skipping (6 m), double limb ankle hops (6 reps), split squat jump (5 reps), and standing jump and reach (5 reps). Characteristics of the squat jump (SJ) and the countermovement jump (CMJ) (jump height, maximal force before take-off, and average power) were determined by force platform (Quattro Jump, version 1.04; Kistler Instrument AG, Winterthur, Switzerland). Jump height was determined as the center of mass displacement, calculated from the recorded force and body mass. Subjects began the SJ at 90 degrees of knee flexion, avoiding any downward movement, and performed a vertical jump by ballistically extending their legs. In the CMJ, they began from the upright position (0 degree knee angle), making a downward movement to 90° of knee flexion and simultaneously beginning the push-off into full extension of the legs. We recorded the largest of three jumps for each test.

##### Force–velocity test

Prior to sprint testing, each subject performed a 5 min warm up, which consisted of 3 min of running, change of direction activities and dynamic stretching. The last 2 min of the warmup, consisted of two practice sprint starts of 3–4 s duration. Force–velocity measurements for the lower limbs were performed on a Monark cycle ergometer (model 894 E, Monark Exercise AB, Vansbro, Sweden) (Chelly et al., 2010a). In brief, the instantaneous maximal pedaling velocity during a 7 s all-out sprint was determined for each braking force, and the participant was judged to have reached peak power (Wpeak) if an additional load induced a decrease in power output. The upper limbs were tested using an appropriately modified cycle ergometer (Hermassi et al., 2010). The parameters measured included Wpeak, the maximal pedaling force for the upper and the lower limbs and the maximal pedaling velocity for the upper and lower limbs (Chelly et al., 2010b). The upper limb tests began with a braking force equal to 1.5% of the participant's body mass (Chelly et al., 2010b). After a 5 min recovery, the braking was increased in sequence to 2, 3, 4, 5, 6, 7, 8, and 9% of the individual's body mass. For more details, (see Chelly et al., 2010b).

##### 30 m sprint

Prior to each session, athletes performed a warm-up for 5 min of running, dynamic activities and stretching. Subjects ran 40 m from a standing position, with the front foot 0.2 m behind the starting photocell beam. Times at 5, 15, and 30 m were recorded by paired photocells (Microgate, Bolzano, Italy) that were located 1 m above the ground at the start and finish lines. Three trials were separated by 6–8 min of recovery, with the fastest times being used in analyses.

##### Handball throwing

Handball-specific throwing velocity was evaluated by a 3-step running throw and a jump throw. A 15 min warm-up was completed prior to test, that included jogging, lateral displacement, dynamic stretching, and jumping. For testing, participants threw a standard handball (mass 480 g, circumference 58 cm) toward the upper right corner of the goal positioned 9 m from each subject. Each individual continued until three throws had been recorded, to a maximum of three sets of three consecutive throws. A 1 to 2 min rest was allowed between sets and 10–15 s between individual throws. In the jump-throw, players took a preparatory three-step run before jumping vertically and releasing the ball while in the air. In the running throw, a preparatory run of three regular steps was made before releasing the ball. Throwing time was recorded with an accuracy of 1 ms, using a digital video camera (HVR to A1U DV Camcorder; Sony, Tokyo, Japan) positioned 2 m above and perpendicular to the ball release. Data processing software (Regavi & Regressi, Micrelec, Coulommiers, France) converted handball displacement to velocities. The validity of the camera and data processing software was previously verified (Chelly et al., 2009). The throw with the greatest average velocity was selected for analysis. The test-retest coefficient of variation for handball throwing times was 1.9%.

##### The Yo-Yo intermittent recovery test level 1

The Yo-Yo-IR1-test was performed as outlined by Krustrup et al. (2003). A standardized warm up prior to testing was comprised of 10 min of low-intensity running, which involved basic run-throughs at an increasing tempo, dynamic stretching, and change of direction activities. For testing, 20-m shuttle runs were performed at increasing velocities, with 10 s of active recovery (2 × 5 m of jogging) between runs until the participant was exhausted. The test itself was considered completed if the participant twice failed to reach the finish line in time (objective evaluation) or felt unable to complete another shuttle at the dictated speed (subjective evaluation). The total distance covered during the Yo-Yo-IR1-test was considered as the test “score” (Castagna et al., 2006). The mean heart rate during the first 10 min of recovery following the Yo-Yo-IR1-test was measured and calculated (Yo-Yo heart rate R_0_-R_10_). The Yo-Yo-IR1-test is known to have a coefficient of variation (CV) of 3.6% with an ICC of 0.94 (Krustrup et al., 2003).

#### Statistical Analyses

Descriptive statistics [mean, standard deviation (SD), minimum, maximum, and 95% confidence intervals (95% CI)] were ascertained for all variables. Mean differences of anthropometric and performance parameters between performance levels (first league vs. second league) and playing positions (pivots vs. wings vs. backs) were tested using a two-factor univariate general linear model (Bortz, 1999). Differences between means were considered statistically significant if *p*-values were <0.05 and partial eta-squared ($\eta_{\text{p}}^{2}$) values were >0.20 (Richardson, 2011). Because of the small number of cases (e.g., position-specific analysis) and in order to avoid an overestimation of mean differences, the decision of significance were made primarily based on $\eta_{\text{p}}^{2}$ values and only for the dependent variable league membership. Statistical analysis was performed using SPSS version 25.0 for Windows (SPSS Inc., Chicago, IL, USA).

## Results

### Anthropometric Data

The first league players (20.4 ± 0.88 years) were significantly younger than the second league players (21.3 ± 1.61 years; *p* = 0.012 and $\eta_{\text{p}}^{2}$ = 0.181; Table 1).

**Table 1:** Demographic and anthropometric characteristics in relation to playing positions and competitive level.

**Playing positions**	**Age [years]**	**Body height [m]**	**Body mass [kg]**	**BMI [kg/m^**2**^]**	**Body fat [%]**
	**Mean ± SD** **(95% CI)**	**Mean ± SD** **(95% CI)**	**Mean ± SD** **(95% CI)**	**Mean ± SD** **(95% CI)**	**Mean ± SD** **(95% CI)**
**FIRST HANDBALL LEAGUE (*****n*** **= 20**)					
Pivots (*n =* 5)	20.0 ± 0.71 (19.2–20.9)	1.87 ± 0.06 (1.82–1.92)	95.4 ± 11.1 (88.3–103)	27.3 ± 2.01 (25.4–29.2)	16.0 ± 1.89 (14.3–17.8)
Wings (*n =* 7)	20.4 ± 0.98 (19.7–21.2)	1.76 ± 0.04 (1.72–1.80)	73.9 ± 4.22 (67.8–79.9)	23.9 ± 1.99 (22.3–25.5)	11.6 ± 1.67 (10.0–13.1)
Backs (*n =* 8)	20.5 ± 0.93 (19.8–21.2)	1.89 ± 0.05 (1.85–1.93)	93.4 ± 7.33 (87.7–99.0)	26.2 ± 2.04 (24.7–27.7)	15.1 ± 2.09 (13.7–16.5)
**SECOND HANDBALL LEAGUE (*****n*** **= 18)**					
Pivots (*n =* 4)	22.3 ± 1.71 (20.5–24.0)	1.87 ± 0.06 (1.81–1.93)	97.3 ± 9.39 (91.0–104)	27.7 ± 1.89 (25.8–29.7)	16.0 ± 1.89 (13.8–18.2)
Wings (*n =* 6)	21.0 ± 1.41 (19.6–22.4)	1.79 ± 0.07 (1.74–1.84)	71.5 ± 4.59 (66.4–76.6)	22.4 ± 1.25 (20.9–24.0)	12.9 ± 1.27 (11.1–14.7)
Backs (*n =* 8)	21.1 ± 1.73 (19.9–22.4)	1.90 ± 0.05 (1.85–1.94)	93.3 ± 4.59 (88.8–97.7)	26.0 ± 2.12 (24.7–27.4)	15.1 ± 2.09 (13.6–16.7)
ANOVA (*p*; $\eta_{\text{p}}^{2}$)	***p*** **= 0.012;** $\eta_{\text{p}}^{2}$ **= 0.181**	*p =* 0.478; $\eta_{\text{p}}^{2}$ = 0.016	*p =* 0.927; $\eta_{\text{p}}^{2}$ = 0.000	*p =* 0.526; $\eta_{\text{p}}^{2}$ = 0.013	*p =* 0.522; $\eta_{\text{p}}^{2}$ = 0.013

*CI, confidence interval. Significant mean differences for the total sample (dependent variable: league) are highlighted in bold*.

Independent from competition level, wings showed the lowest body mass, body height, body fat, and BMI (Table 1). The same was observed for muscle volume (Table 2). The pivots' thigh volume of the second league players was found to have the lowest values (6.58 ± 1.80 l).

**Table 2:** Muscle volumes in relation to playing positions and competitive level.

**Playing positions**	**Muscle volume lower limb [l]**	**Muscle volume upper limb [l]**	**Thigh volume [l]**	**Cross sectional area [cm^**2**^]**
	**Mean ± SD** **(95% CI)**	**Mean ± SD** **(95% CI)**	**Mean ± SD** **(95% CI)**	**Mean ± SD** **(95% CI)**
**FIRST HANDBALL LEAGUE (*****n*** **= 20)**				
Pivots (*n =* 5)	11.9 ± 2.07 (10.4–13.3)	4.06 ± 0.27 (3.41–4.72)	8.00 ± 1.72 (6.58–9.42)	200 ± 2.41 (195–204)
Wings (*n =* 7)	9.07 ± 1.70 (7.87–10.3)	3.06 ± 0.49 (2.51–3.61)	6.58 ± 1.83 (5.38–7.78)	180 ± 6.60 (177–184)
Backs (*n =* 8)	11.1 ± 0.76 (10.0–12.3)	4.05 ± 0.96 (3.53–4.56)	8.42 ± 0.97 (7.30–9.55)	201 ± 2.67 (197–204)
**SECOND HANDBALL LEAGUE (*****n =*** **18)**				
Pivots (*n =* 4)	11.2 ± 1.70 (9.80–12.5)	3.27 ± 0.18 (2.97–3.56)	6.58 ± 1.80 (5.01–8.15)	199 ± 1.29 (197–200)
Wings (*n =* 6)	9.51 ± 1.53 (8.40–10.6)	2.97 ± 0.44 (2.73–3.22)	7.12 ± 1.50 (5.84–8.40)	174 ± 2.43 (172–175)
Backs (*n =* 8)	10.6 ± 0.75 (9.66–11.6)	3.17 ± 0.14 (2.96–3.38)	7.89 ± 1.28 (6.79–9.00)	199 ± 1.13 (198–200)
ANOVA (*p*; $\eta_{\text{p}}^{2}$)	*p =* 0.574; $\eta_{\text{p}}^{2}$ = 0.010	***p*** **= 0.003;** $\eta_{\text{p}}^{2}$ **= 0.248**	*p =* 0.354; $\eta_{\text{p}}^{2}$ = 0.027	*p =* 0.010; $\eta_{\text{p}}^{2}$ = 0.192

*CI, confidence interval. Significant mean differences for the total sample (dependent variable: league) are highlighted in bold*.

The anthropometric data also revealed significantly (*p* = 0.003 and $\eta_{\text{p}}^{2}$ = 0.248) less muscle volume of upper limb for second league players (3.13 ± 0.29 l) than for first league players (3.71 ± 0.82 l). The cross-sectional area for the first league players (193 ± 10.6 cm^2^) was also larger than the second league players (190 ± 12.4 cm^2^; *p* = 0.003, $\eta_{\text{p}}^{2}$ = 0.248). There were no other significant anthropometric differences. In both leagues, the players with the highest body mass, BMI and body fat were pivots, while the backs were the tallest athletes in the whole sample (Table 1).

### Performance Data

**Figure 1:**
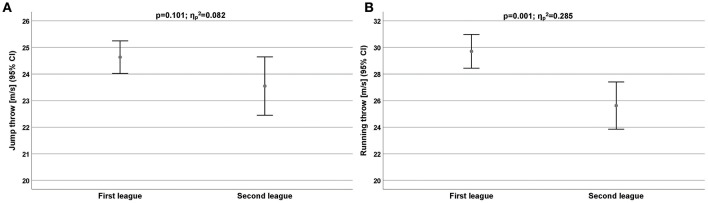
**(A,B)** Throwing performance measured by jump throw velocity **(A)** and running throw velocity **(B)** for all players depend on competitive level. CI, Confidence interval.

The throwing velocity data (Table 3) revealed that the first league players have a higher throwing velocity than the second league players (Figures 1A,B). But the difference was only significant for the running throw ($\eta_{\text{p}}^{2}$ = 0.285; Figure 1B).

**Table 3:** Throwing velocity and sprinting performance depend on the type of throw (jump throw, running throw) and different distance (15 m, 30 m), playing positions and competitive level.

**Playing positions**	**Throwing velocity [m/s]**		**Sprinting performance [s]**	
	**Jump throw**	**Running throw**	**15 m**	**30 m**
	**Mean ± SD** **(95% CI)**	**Mean ± SD** **(95% CI)**	**Mean ± SD** **(95% CI)**	**Mean ± SD** **(95% CI)**
**FIRST HANDBALL LEAGUE (*****n*** **= 20)**				
Pivots (*n =* 5)	24.8 ± 1.61 (23.5–26.1)	30.4 ± 2.10 (27.7–33.0)	2.47 ± 0.22 (2.30–2.64)	4.59 ± 0.17 (4.42–4.77)
Wings (*n =* 7)	24.9 ± 1.50 (23.8–26.0)	29.7 ± 2.59 (27.4–31.9)	2.49 ± 0.17 (2.34–2.63)	4.06 ± 0.10 (3.92–4.21)
Backs (*n =* 8)	24.3 ± 1.01 (23.3–25.3)	29.3 ± 3.35 (27.2–31.4)	2.68 ± 0.17 (2.54–2.81)	4.59 ± 0.24 (4.45–4.72)
**SECOND HANDBALL LEAGUE (*****n*** **= 18)**				
Pivots (*n =* 4)	26.3 ± 2.37 (24.7–27.9)	27.4 ± 3.17 (23.6–31.3)	2.64 ± 0.08 (2.18–3.09)	4.96 ± 0.18 (4.72–5.20)
Wings (*n =* 6)	21.8 ± 1.57 (20.5–23.1)	25.8 ± 3.65 (22.6–28.9)	2.50 ± 0.19 (2.13–2.87)	3.83 ± 0.18 (3.63–4.02)
Backs (*n =* 8)	23.5 ± 0.78 (22.4–24.6)	24.6 ± 3.78 (21.9–27.4)	3.14 ± 0.60 (2.82–3.46)	4.54 ± 0.27 (4.37–4.71)
ANOVA (*p*; $\eta_{\text{p}}^{2}$)	*p =* 0.101; $\eta_{\text{p}}^{2}$ = 0.082	***p*** **= 0.001;** $\eta_{\text{p}}^{2}$ **= 0.285**	*p =* 0.054; $\eta_{\text{p}}^{2}$ = 0.111	*p =* 0.662; $\eta_{\text{p}}^{2}$ = 0.006

*CI, confidence interval. Significant mean differences for the total sample (dependent variable: league) are highlighted in bold*.

The pivots were the only subsample with a lower throwing velocity in the first league than in the second league (24.8 ± 1.61 vs. 26.3 ± 2.37 m/s).

**Figure 2:**
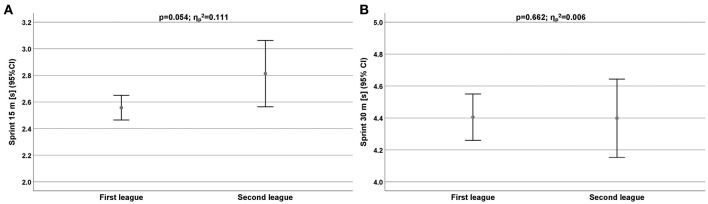
**(A,B)** Sprinting performance measured by 15 m **(A)** and 30 m **(B)** for all players depend on competitive level. CI, Confidence interval.

Similar results were provided for sprinting performance (Table 3). Except the wings (4.06 ± 0.10 s vs. 3.83 ± 0.18 s), and all first league players were faster than the second league players (Figure 2A).

There was a significant difference in running performance for the 15 m sprint ($\eta_{\text{p}}^{2}$ = 0.111; Figure 2B).

**Figure 3:**
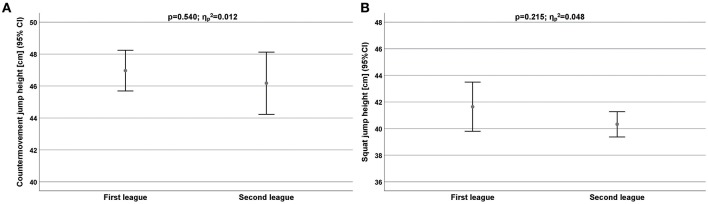
**(A,B)** Jumping performance measured by countermovement jump **(A)** and squat jump **(B)** for all players depend on competitive level. CI, Confidence interval.

Jumping performance (SJ, CMJ) was also found to be higher among the first league players compared to second league players (Table 4; Figures 3A,B).

**Table 4:** Squat jump performance depend on playing positions and competitive level.

**Playing positions**	**Squat jumping performance**			
	**Height [cm]**	**Power [W/kg]**	**Force [N]**	**Velocity [m/s]**
	**Mean ± SD** **(95% CI)**	**Mean ± SD** **(95% CI)**	**Mean ± SD** **(95% CI)**	**Mean ± SD** **(95% CI)**
**FIRST HANDBALL LEAGUE (*****n*** **= 20)**				
Pivots (*n =* 5)	41.2 ± 2.85 (37.3–45.1)	39.6 ± 3.10 (37.2–42.0)	1,871 ± 150 (1,596–2,145)	1.92 ± 0.19 (1.78–2.05)
Wings (*n =* 7)	42.5 ± 3.65 (39.2–45.8)	40.0 ± 1.97 (38.0–42.1)	1,609 ± 178 (1,376–1,841)	1.90 ± 0.10 (1.79–2.02)
Backs (*n =* 8)	41.2 ± 5.03 (38.1–44.3)	38.4 ± 2.64 (36.5–40.3)	1,942 ± 407 (1,725–2,160)	1.73 ± 0.14 (1.62–1.84)
**SECOND HANDBALL LEAGUE (*****n*** **= 18)**				
Pivots (*n =* 4)	39.9 ± 2.98 (37.7–42.0)	24.5 ± 5.14 (17.4–31.6)	1,889 ± 281 (1,621–2,157)	2.50 ± 0.06 (2.42–2.57)
Wings (*n =* 6)	40.1 ± 1.96 (38.3–41.8)	24.9 ± 3.02 (19.1–30.7)	1,727 ± 277 (1,508–1,946)	2.51 ± 0.05 (2.45–2.58)
Backs (*n =* 8)	40.8 ± 1.37 (39.3–42.3)	28.8 ± 8.79 (23.8–33.8)	1,725 ± 217 (1,535–1,914)	2.52 ± 0.10 (2.46–2.57)
ANOVA (*p*; $\eta_{\text{p}}^{2}$)	*p =* 0.215; $\eta_{\text{p}}^{2}$ = 0.048	***p*** **< 0.001;** $\eta_{\text{p}}^{2}$ **= 0.670**	*p =* 0.771; $\eta_{\text{p}}^{2}$ = 0.003	***p*** **< 0.001;** $\eta_{\text{p}}^{2}$ **= 0.900**

*CI, confidence interval. Significant mean differences for the total sample (dependent variable: league) are highlighted in bold*.

The difference was higher in SJ [two significant differences: power ($\eta_{\text{p}}^{2}$ = 0.670), velocity ($\eta_{\text{p}}^{2}$ = 0.900)] than in CMJ. The second league players (2.51 ± 0.07 m/s) showed a significantly higher SJ velocity than the first league players (1.84 ± 0.16 m/s). In contrast, there was a difference in SJ power ($\eta_{\text{p}}^{2}$ = 0.670) but in favor of the first league players (39.3 ± 2.52 vs. 26.5 ± 6.59 W/kg). Identical results were calculated in both leagues for SJ force ($\eta_{\text{p}}^{2}$ = 0.003).

The CMJ performance was the only performance that showed no significant differences in all parameters based on league membership (Table 5).

**Table 5:** Countermovement jump performance depend on playing positions and competitive level.

**Playing positions**	**Countermovement jumping performance**			
	**Height [cm]**	**Power [W/kg]**	**Force [N]**	**Velocity [m/s]**
	**Mean ± SD** **(95% CI)**	**Mean ± SD** **(95% CI)**	**Mean ± SD** **(95% CI)**	**Mean ± SD** **(95% CI)**
**FIRST HANDBALL LEAGUE (*****n*** **= 20)**				
Pivots (*n =* 5)	46.5 ± 2.36 (43.9–49.1)	24.7 ± 4.49 (20.8–28.5)	1,930 ± 275 (1,698–2,161)	2.07 ± 0.16 (1.96–2.18)
Wings (*n =* 7)	48.1 ± 3.16 (45.9–50.3)	27.0 ± 3.74 (23.8–30.3)	1,507 ± 157 (1,312–1,703)	2.07 ± 0.08 (1.97–2.16)
Backs (*n =* 8)	46.3 ± 2.57 (44.3–48.4)	26.3 ± 4.05 (23.3–29.3)	1,626 ± 286 (1,443–1,809)	2.03 ± 0.11 (1.95–2.12)
**SECOND HANDBALL LEAGUE (*****n*** **= 18)**				
Pivots (*n =* 4)	45.8 ± 3.81 (41.6–50.0)	25.5 ± 3.99 (22.3–28.7)	1,935 ± 297 (1,674–2,196)	1.95 ± 0.11 (1.67–2.22)
Wings (*n =* 6)	47.8 ± 4.16 (44.3–51.2)	28.3 ± 1.44 (25.7–30.9)	1,550 ± 206 (1,338–1,763)	1.98 ± 0.14 (1.76–2.20)
Backs (*n =* 8)	45.2 ± 3.92 (42.2–48.2)	25.9 ± 3.28 (23.6–28.1)	1,679 ± 245 (1,494–1,863)	2.09 ± 0.35 (1.90–2.28)
ANOVA (*p*; $\eta_{\text{p}}^{2}$)	*p =* 0.540; $\eta_{\text{p}}^{2}$ = 0.012	*p =* 0.647; $\eta_{\text{p}}^{2}$ = 0.007	*p =* 0.684; $\eta_{\text{p}}^{2}$ = 0.005	*p =* 0.441; $\eta_{\text{p}}^{2}$ = 0.019

*CI, confidence interval*.

**Figure 4:**
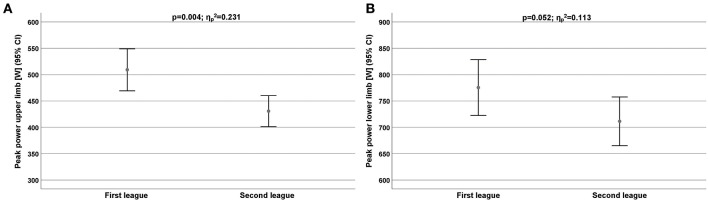
**(A,B)** Peak power performance for upper limb **(A)** and lower limb **(B)** for all players depend on competitive level. CI, Confidence interval.

The strength data (Table 6) showed that the first league players were significantly stronger than the second league players. The difference of performance levels was higher in the upper limb (Figure 4A) than in the lower (Figure 4B) limb ($\eta_{\text{p}}^{2}$ = 0.231 vs. $\eta_{\text{p}}^{2}$ = 0.113).

**Table 6:** Power peak performance of upper and lower limb and aerobic capacity measuring in Yo-Yo-IR1 depend on playing positions and competitive level.

**Playing positions**	**Power peak performance [W]**		**Aerobic capacity**
	**Upper limb**	**Lower limb**	**Yo-Yo-IR 1 [m]**
	**Mean ± SD** **(95% CI)**	**Mean ± SD** **(95% CI)**	**Mean ± SD** **(95% CI)**
**FIRST HANDBALL LEAGUE (*****n*** **= 20)**			
Pivots (*n =* 5)	512 ± 108 (429–595)	781 ± 118 (677–885)	1.656 ± 78.0 (1.585–1.727)
Wings (*n =* 7)	486 ± 56.7 (416–556)	722 ± 58.6 (634–809)	1.869 ± 68.2 (1.809–1.929)
Backs (*n =* 8)	528 ± 96.6 (462–593)	819 ± 136 (737–902)	1.670 ± 79.3 (1.614–1.726)
**SECOND HANDBALL LEAGUE (*****n*** **= 18)**			
Pivots (*n =* 4)	413 ± 41.6 (347–479)	712 ± 93.1 (583–782)	1.850 ± 100 (1.674–2.026)
Wings (*n =* 6)	447 ± 50.6 (394–501)	686 ± 57.2 (605–768)	2.107 ± 136 (1.963–2.251)
Backs (*n =* 8)	427 ± 74.5 (381–474)	745 ± 82.4 (674–815)	1.760 ± 203 (1.636–1.885)
ANOVA (*p*; $\eta_{\text{p}}^{2}$)	***p*** **= 0.004;** $\eta_{\text{p}}^{2}$**=0.231**	*p =* 0.052; $\eta_{\text{p}}^{2}=$0.113	***p*** **< 0.001;** $\eta_{\text{p}}^{2}$**=0.348**

*CI, confidence interval. Significant mean differences for the total sample (dependent variable: league) are highlighted in bold*.

In the first league, the backs are consistently the strongest cohort compared to the other positions. In contrast, in the second league, the wings (upper limb) and the backs (lower limb) showed the highest power peak performance.

**Figure 5:**
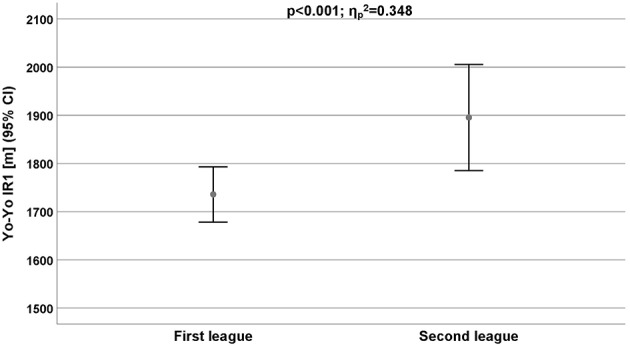
Endurance performance (aerobic capacity) measured by Yo-Yo IR1 for all players depend on competitive level. CI, Confidence interval.

The aerobic capacity (Yo-Yo-IR1) was the only performance that displayed a significantly higher performance level ($\eta_{\text{p}}^{2}$ = 0.348) of the second league players compared to the first league players (Table 6; Figure 5).

In all positions, the second league players showed a longer distance in the Yo-Yo-IR 1 test than the first league players. Independent from the league membership, the wings achieved the longest test distances within the Yo-Yo-IR1 test (first league: 1.869 ± 68.2 m; second league: 2.107 ± 136 m).

## Discussion

Profiling studies in comparable designs like this one have been used in a variety of sports in an attempt to develop reference data and to standardize test procedures. Two major conclusions emerge from the present study: (1) performance characteristics (peak power, throwing velocity, aerobic capacity) differed significantly among leagues in male handball players; (2) body height was different between groups with the first league players being significantly taller than second league players; and (3) different results were provided for sprinting performance however, the wings, and all first league players were faster than the second league players.

The anthropometric data revealed significantly (*p* = 0.003 and $\eta_{\text{p}}^{2}$ = 0.248) less muscle volume for second league players (3.13 ± 0.29 l) than for first league players (3.71 ± 0.82 l). For all other anthropometric parameters (Tables 1, 2), we didn't find any significant difference. In both leagues, the players with the highest body mass, BMI, and body fat were pivots, while the backs were the tallest athletes in the whole sample (Table 1). Earlier studies documented the age (23.1–31.3 years), body height (1.82–1.91 m), and body mass (82.2–95.6 kg) of elite male European handball players (Srhoj et al., 2002; Gorostiaga et al., 2006; Šibila and Pori, 2009; Povoas et al., 2012; Massuca et al., 2014). The physical characteristics reported in these articles were similar to averages observed in the present study, as well as those from the study of German first and second Division teams by Krüger et al. (2014). Although, it should be noted that the tallest sub-group (backs, *n* = 25) were over-represented in the group examined in this study.

In our study the leg muscles of players of the first league were relatively well-developed, despite an average body mass of only 87 kg. The total leg volume of 10.6 l exceeded the value of players of the second league 10.4 l with a body mass average of 86 kg. The leg muscle volume found in the present study was less than that seen by Hermassi et al. (2011) of elite handball players, but was similar to those reported by Hermassi et al. (2018) and Chelly et al. (2010a) of male handball players. In addition, the total leg volume exceeded the value previously estimated for young adult males (Shephard et al., 1988a,b) and soccer players (Chelly et al., 2009).

The thigh muscle volume of First Handball League found in the present study was less than that seen by Aloui et al. (2018) of junior's handball players, but was similar to those reported by Hermassi et al. (2011) male handball players. The thigh muscle volume of First Handball League players was significantly larger than the second league players. In fact, the First Handball League, the backs (8.42 ± 0.97 l) had higher values than pivots (8.00 ± 1.72 l) and wings (6.58 ± 1.83 l).

The cross-sectional area of the thigh in First Tunisia Handball League players were significantly higher than the second league players but similar to those reported by Hermassi et al. (2011) and less than that seen by Aloui et al. (2018) in junior's handball players. In fact, the First Tunisia Handball League, the backs (201 ± 2.67 cm^2^) achieved higher values of thigh muscle than pivots (200 ± 2.41 cm^2^) and wings (180 ± 6.60 cm^2^). The cross-sectional area, differ significantly between level and ranking of players reflected neuronal adaptations, a well-accepted response to regime of training (Hermassi et al., 2011).

In our study muscle volume of the upper limb in players of the first league were relatively more developed than the value of players of the second league. Muscle volume of upper limb First Tunisia Handball League found in the present study was higher than that seen by (Hermassi et al., 2011) of elite handball players, but was similar to those reported by Aloui et al. (2018) and Chelly et al. (2010a) of male handball players. In fact, the First Tunisia Handball League, pivots (4.06 ± 0.27 l) achieved higher values than backs (4.05 ± 0.96 l) and wings (3.06 ± 0.49 l).

Power considered as determinant factors of success in elite level of handball. Schmidtbleicher (1992) defined power as the greatest impulse the neuromuscular system could produce in a given time. The absolute peak power of First Tunisia Handball League found in the present study equal to 512 ± 108 W and was less than that seen by Hermassi et al. (2010) of male handball players, and Soccer players (Chelly et al., 2010a) but was similar to those reported by Hermassi et al. (2018) and Hermassi et al. (2011) handball players. Equally, the absolute peak power in the present study was similar to value reported by Chelly et al. (2014) for Elite Juniors handball players. In fact, in the First Tunisia Handball League, the backs (528 ± 96.6 W) achieved lower values of peak power (512 ± 108 W) and wing (486 ± 56.7 W). In terms of the force/velocity test, the average maximal power of French soccer players was 1.021 W, but much of their apparent advantage over the present sample was due to a larger body mass. The absolute peak power of First Handball League found in the present study equal to 819 ± 136 W and was less than that seen by Hermassi et al. (2018) of elite male handball players, and juniors handball players (Aloui et al., 2018) but was similar to those reported by Hermassi et al. (2011) and higher than reported by Chelly et al. (2010a) for soccer players.

The throwing velocity in handball is important to achieve success in the sport because the faster the ball is thrown at the goal, the less time the defenders and the goalkeeper have to save the shot. We observed significant differences of physical performance dependent on competition level (first vs. second league). The throwing velocity data revealed that the first league players have a higher throwing velocity than the second league players. However, the difference was only significantly for running throw ($\eta_{\text{p}}^{2}$ = 0.285). The pivots are the only subsample with a lower throwing velocity in the first league than in the second league (24.8 ± 1.61 vs. 26.3 ± 2.37 m/s). The profile of requirement due to this specific sport can only be sequentially reflected with regard to speed and endurance components. Other studies of elite handball players show a mean throwing velocity of 17.1–22.2 m/s (Gorostiaga et al., 2006; Chelly et al., 2010b; Hermassi et al., 2010, 2011, 2018; Granados et al., 2013; Fieseler et al., 2017). The velocities reached by our handball players are in line with those of the aforementioned studies.

Our results for sprinting performance (15, 30 m) were similar to those for throwing performance with first league players performing better than the second league athletes. Except the wings (4.06 ± 0.10 s vs. 3.83 ± 0.18 s), all first league players were faster than the second league players. The performance difference was significant for sprint 15 m ($\eta_{\text{p}}^{2}$ = 0.111). Normally, elite players should have a higher level of aerobic capacity than second league players in relation to their overall time of training per week. Therefore, the ability to recover and the sprinting performance under load should be better. Haugen et al. (2016) reported substantial faster times of 2.80 s in average for a 20 m sprint than in our study (first league: 3.74 s; third league: 3.49 s).

This difference may be explained by the exclusive assessment compared to the current study, where the sprint was measured during the 15 and 30 m protocol separately. In addition the players evaluated in the study of Haugen et al. (2016) were National team athletes. Therefore, comparability with current data might be limited. Reasons for the higher performance of second league players could be a higher test motivation for these athletes. Also, the higher number of wings in the second league sample could be partially responsible for the better sprint performance.

Additionally, the competitive athletic training in the third league team may be more sufficient than in the first league sample. This hypothesis is in line with the information regarding the first league team, that the main part of the athletic training was conducted in a CrossFit® studio. A sprint performance and technical training of running was lacking in the first league team. Maybe, the strength training in a CrossFit® studio induced fatigue effects for the upper limb and reduced the flexibility of the throwing arm.

The CMJ scores of our players are higher than those previously reported (Hermassi et al., 2011; Chelly et al., 2014). Therefore, we can conclude that our players exhibited similar values of lower body power without taking into consideration the specific playing position. Therefore, jumping performance (SJ, CMJ) was also found to be higher among the first league players compared to second league players. The difference was sharply higher in SJ [two significant differences: power ($\eta_{\text{p}}^{2}$ = 0.670), velocity ($\eta_{\text{p}}^{2}$ = 0.900)] than in CMJ. The second league players (2.51 ± 0.07 m/s) showed a significantly higher SJ velocity than the first league players (1.84 ± 0.16 m/s). In contrast, regarding the SJ power, we also detected a great difference ($\eta_{\text{p}}^{2}$ = 0.670) but in favor of the first league players (39.3 ± 2.52 vs. 26.5 ± 6.59 W/kg). Identical results were calculated in both leagues for SJ force ($\eta_{\text{p}}^{2}$ = 0.003). The CMJ performance was the only performance that showed not significant differences in all parameters based on league membership.

Team handball is characterized by explosive actions performed at high velocities; therefore, success in competition depends essentially on a well-developed muscular strength (Aloui et al., 2018; Hermassi et al., 2018). The higher values of maximum strength and muscle strength provide a clear advantage for maintaining of muscle contractions during the entire match. The strength training and weight training are important for improvement of performance in handball. The strength data showed that the first league players were significantly stronger than the second league players. The difference of performance levels was higher in the upper limb than in the lower limb ($\eta_{\text{p}}^{2}$ = 0.231 vs. $\eta_{\text{p}}^{2}$ = 0.113). In the first league, the backs are consistently the strongest cohort compared to the other positions. In contrast, in the second league, the wings (upper limb) and the backs (lower limb) showed the highest power peak performance.

Thus, similar to other team sports, handball involves activities that require well-developed aerobic and anaerobic qualities. Povoas et al. (2012) reported that during a 60 min match, players covered about 4 km at a mean intensity of 87% of maximal heart rate (HR), demonstrating that about 90% of the total energy expenditure was aerobic (Povoas et al., 2012). Match-analyses have shown that handball involves many intermittent high-intensity activities throughout the game (Šibila and Pori, 2009; Povoas et al., 2012). Therefore, the ability to perform intermittent high-intensity exercise for the entire game, and to recover quickly from high intensity exercise bouts should be considered a logical criterion in team-handball selection, training, and testing. The aerobic capacity (Yo-Yo-IR1) was the only performance that displayed a significant higher performance level ($\eta_{\text{p}}^{2}$ = 0.348) of the second league players compared to the first league players. In all positions, the second league players showed a longer distance in the Yo-Yo-IR 1 test than the first league players. Independent from the league membership, the wings achieved the longest test distances within the Yo-Yo-IR1 test (first league: 1.869 ± 68.2 m; second league: 2.107 ± 136 m).

### Limitation

The main limitation of this study, especially regarding positional subgroups, is the small sample size. Therefore, these data should be interpreted with caution and in comparison with similar investigations. It is evident, that this sample size is not powerful enough to generate statements about anthropometrics and positional success for an entire sport. Furthermore, it might be considered, that the motivation of some first league players were limited. It could be an explanation for the lower performance level of first league players compared to third league players.

## Conclusions

This study provides a detailed analysis of movement patterns in relation to physiological profiles in elite professional handball athletes. These findings provide information for the assessment and evaluation of talents and should help to develop and optimize position training regimes. These findings may also be beneficial in the prevention, evaluation, and treatment of injuries commonly sustained by handball players. In this work, second league players showed a lower performance level regarding throwing, sprinting, and jumping than first league players, which underscores the challenge and difficulty of a valid performance and diagnostic program. Performance capacities clearly differ across playing positions, and coaches should thus develop position-specific training concepts.

However, further profiling of handball players is required before definitive reference data can be presented. Scientists should also be encouraged to study factors that contribute to the progressive deterioration in performance over the course of a game; the optimal tactic in sustaining performance will likely improve endurance specific to the individual's playing position, plus the avoidance of unnecessary rapid movements early in the game. Furthermore, the results should encourage thinking about a sufficient performance diagnostic concept in team handball. Coaches and scientists should recognize the unique nature of training regimen with the different physical profiling level positions and match these to the demands of team handball practice and training prescription.

## Data Availability

All datasets generated for this study are included in the manuscript and/or the supplementary files.

## Author Contributions

SH and RS: formal analysis and methodology. SH: investigation, project administration, and supervision. KL and RS: writing–original draft. SH, KL, and RS: writing–review and editing.

### Conflict of Interest Statement

The authors declare that the research was conducted in the absence of any commercial or financial relationships that could be construed as a potential conflict of interest.
